# *EZH2* mutations increase the heterogeneity of chromatin states in lymphoma

**DOI:** 10.1371/journal.pbio.3003211

**Published:** 2025-06-13

**Authors:** Daniel Holoch, Raphaël Margueron

**Affiliations:** Institut Curie, INSERM U934/CNRS UMR 3215, Paris Sciences et Lettres Research University, Sorbonne University, Paris, France

## Abstract

This Primer discusses a recent study using simultaneous measurements of histone modifications in single cells to reveal that *EZH2* gain-of-function mutations profoundly reprogram chromatin states in B-cell lymphoma, while also increasing their cell-cell heterogeneity.

## Teaser

A recent study in *PLoS Biology* used simultaneous measurements of histone modifications in single cells to reveal that *EZH2* gain-of-function mutations profoundly reprogram chromatin states in B-cell lymphoma, while also increasing their cell–cell heterogeneity.

The transcription programs that underpin differentiation and cell identity depend on the concerted action of transcription factors and chromatin modifiers. Oncogenesis involves a weakening of cell identity and, as such, is very frequently associated with alterations in chromatin regulators [1].

This is especially striking in germinal-center-B cell-like diffuse large B cell lymphomas (GCB-DLBCL) and follicular lymphomas (FL), which both feature mutations in key histone-modifying enzymes in more than half of patients [2,3]. These lymphomas arise within germinal centers (GCs), where activated B cells normally undergo affinity maturation as they prepare to differentiate into plasma cells and memory B cells. The GC reaction involves cycles of somatic hypermutation and clonal expansion, processes that create inherent vulnerability to malignant transformation [2,3].

Normal GC formation and function require transient upregulation of EZH2, a conserved histone H3 lysine 27 (H3K27) methyltransferase. In GC B cells, EZH2 targets key regulators of proliferation checkpoints and differentiation programs whose temporary suppression is critical for successful affinity maturation [4,5]. Subsequent failure to reduce EZH2 activity, however, could drive cells toward lymphomagenesis. Indeed, approximately 1 in 4 FL and GCB-DLBCL tumors present heterozygous gain-of-function (GOF) mutations in *EZH2*, resulting in elevated H3K27 trimethylation (H3K27me3) [6,7]. This excess EZH2 activity has been linked to aberrant repression of genes required for differentiation accompanied by GC hyperplasia and transformation [4], although the underlying mechanism remains incompletely understood.

Another histone modifier recurrently mutated in GC-derived lymphomas is KMT2D, which monomethylates H3K4 [2,3]. In mouse models, such loss-of-function *KMT2D* mutations obstruct differentiation and increase proliferation of GC B cells, thus promoting the emergence of lymphomas [8,9]. Intriguingly, mutations in *KMT2D* and *EZH2*, as well as loss-of-function mutations in the gene encoding the histone acetyltransferase *CREBBP*, are often found in combination in FL and GCB-DLBCL, indicating that they confer partly non-overlapping advantages to tumor cells.

While several studies have investigated the contributions of individual histone modifier alterations to lymphomagenesis, how these different mutations interact when they co-occur is not well understood. The extent to which disruption of these enzymes perturbs the chromatin landscape more generally, through the complex network of crosstalk with other pivotal histone modifications, is also unclear. Finally, whether these mutations are uniform or variable in their impact on histone modification profiles and gene expression programs across different cells is an underexplored question with important clinical implications. Indeed, intercellular heterogeneity plays a key role in tumor adaptation and evolution, and achieving a precise understanding of a tumor’s constituent cellular phenotypes has the potential to dramatically improve the efficacy of therapeutic interventions.

**Fig 1:**
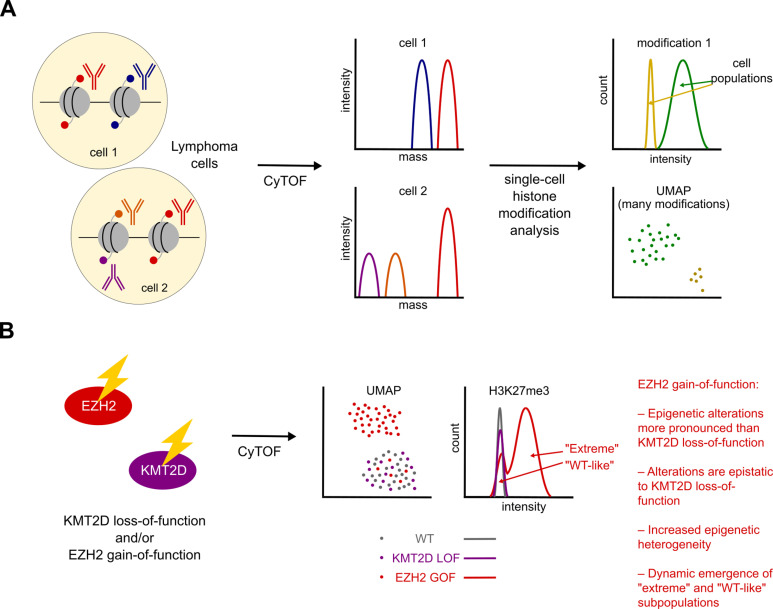
Cytometry by Time of Flight reveals epistasis and heterogeneity. (A) In Cytometry by Time of Flight (CyTOF), cells are stained with metal-conjugated antibodies, here mainly targeting modified histones, and epitope abundance is then quantified in multiplex in large numbers of individual cells. The data can be analyzed mark by mark, as well as across all the marks using dimensionality reduction approaches, to reveal subpopulations and assess heterogeneity. (B) Griess and colleagues explore the consequences of *KMT2D* null and *EZH2* gain-of-function mutations on histone modification profiles in lymphoma cells using CyTOF. They discover that *EZH2* alterations are epistatic to *KMT2D* loss of function (see text) and increase both the epigenomic and transcriptomic heterogeneity of the cells. They identify “extreme” and “WT-like” subpopulations of *EZH2*-mutant cells that form dynamically and show distinct responses to small-molecule inhibitors.

In a new study in *PLoS Biology*, Griess and colleagues sought to address all these aspects of histone modifier function in lymphomagenesis. To do so, they applied a single-cell methodology, Cytometry by Time of Flight (CyTOF), to quantify the levels of 16 different histone modifications and several other markers simultaneously in large numbers of GCB-DLBCL cells [10]. Then, using dimensionality reduction and other analyses, they extracted patterns of co-variation among histone modifications and classified individual cells into clusters based on their overall profiles of histone mark abundance (Fig 1A). This approach allowed the authors to examine the detailed impact of recurrent *KMT2D* and *EZH2* mutations on the histone modification landscape and its cell-cell variability, both in patient-derived xenografts and isogenic DLBCL cell lines.

The data reveal that impaired KMT2D and enhanced EZH2 activity have quite distinct effects on the lymphoma epigenome. The impact of a *KMT2D* null mutation is relatively mild, with little change in H3K4me1 itself and instead modest increases in H3K36me2 and H4K16ac. By contrast, an *EZH2* GOF mutation causes a substantial shift toward H3K27me3 at the expense of H3K27me2, as expected, but also a sharp loss of H3K9me2. Strikingly, combining these mutations produces the same CyTOF profile as *EZH2* GOF alone, indicating that *EZH2* mutations are epistatic to *KMT2D* loss in DLBCL. Further supporting this notion, the authors observe expression changes in a small number of genes in *KMT2D*-null cells, but these are not recapitulated in *EZH2*-GOF/*KMT2D* double-mutant cells. Instead, the double-mutant transcriptome matches the extensive alterations seen in *EZH2*-GOF single-mutant cells. Thus, the *EZH2* GOF mutation induces comparatively drastic changes and exhibits epistasis over *KMT2D* loss of function.

In addition to these properties, the authors’ powerful single-cell approach enabled them to uncover another distinctive feature of *EZH2* GOF DLBCL cells, which is their markedly increased cell-cell heterogeneity in histone modification profiles compared to *KMT2D*-mutant or unmodified counterparts. This is evident both from defined metrics and from their segregation into distinct clusters in dimensionality reduction plots. A clear majority of cells form a population with strong attributes of the *EZH2*-mutant epigenomic profile, termed “extreme” by the authors, while a smaller “WT-like” group clusters with *EZH2*-WT cells (Fig 1B).

Remarkably, the authors could show through subcloning experiments that this bimodal behavior reappears in virtually every clonal population, suggesting that cells are able to shift dynamically between these distinct states. Given that the “WT-like” cells are not as sensitive to EZH2 inhibition as the “extreme” subpopulation, this transition capability raises important implications for targeted therapy. Indeed, *EZH2*-mutant tumors show encouraging but incomplete responses to EZH2 inhibitors in the clinic. The authors’ findings suggest that this limitation might be attributable to “WT-like” cells, for which complementary treatment strategies will need to be devised.

Beyond these novel insights into the impact of *EZH2* and *KMT2D* mutations at the single-cell level in DLBCL, the authors also demonstrated the more general statistical power of these types of datasets by developing new computational analysis pipelines. Thoroughly exploiting their simultaneous measurements of abundance of many histone modifications in many individual cells, they extracted a network model that infers the directionality of causal relationships between changes in different modifications. The complexity of the crosstalk between histone modifications can make such links otherwise difficult to discern, therefore making these new approaches uniquely valuable.

An important goal for future studies will be to determine the locally acting mechanisms that underlie these relationships in global levels of histone marks, especially in the context of lymphomas carrying mutations in histone modifiers. Which molecular encounters along the chromatin can explain the profound shifts and increased heterogeneity revealed in this work? The epistasis relationship uncovered here also raises questions regarding the advantage for lymphomas of simultaneous *KMT2D* and *EZH2* mutations, which future investigations will help clarify. This study’s findings open several new research directions that promise to yield even more advances in the years ahead.

## Abbreviations

CyTOF: Cytometry by Time of Flight
FL: follicular lymphomas
GCs: germinal centers
GCB-DLBCL: germinal-center-B cell-like diffuse large B cell lymphomas
GOF: gain-of-function
H3K27: histone H3 lysine 27
H3K27me3: H3K27 trimethylation
